# A pregnant woman who experienced auditory hallucinations concurrent with hyperemesis gravidarum: A case report

**DOI:** 10.1002/npr2.12206

**Published:** 2021-09-02

**Authors:** Hiroaki Okayasu, Norio Yasui‐Furukori, Kazutaka Shimoda

**Affiliations:** ^1^ Department of Psychiatry Dokkyo Medical University Mibu Japan

**Keywords:** auditory hallucination, dopamine antagonist, female sex hormone, hyperemesis gravidarum, pregnancy

## Abstract

We report the case of a pregnant woman who experienced auditory hallucinations only while suffering from hyperemesis gravidarum. To the best of our knowledge, the present report is the first report of a case of obvious auditory hallucinations and hyperemesis gravidarum at the same time in a pregnant woman who had not been diagnosed with any psychiatric disorder. The patient was a 24‐year‐old pregnant woman with no history of psychiatric disorder. Two years prior to this admission, she became pregnant for the first time, and she was admitted to an obstetrics clinic due to severe hyperemesis gravidarum. She developed mild auditory hallucinations at the same time. After she gave birth, the auditory hallucinations disappeared. When she was 24 years old, she became pregnant again. She suffered from severe hyperemesis gravidarum from the early stage of pregnancy. At 20 weeks of pregnancy, she visited the Department of Psychiatry of our hospital for a detailed psychiatric evaluation and treatment because her moderate auditory hallucinations had relapsed. We administered an antipsychotic agent, perospirone, to treat the auditory hallucinations, which disappeared, although the hyperemesis gravidarum persisted until childbirth. After childbirth, perospirone treatment was discontinued, and her auditory hallucinations did not relapse. The auditory hallucinations may have occurred as a result of complicated biological and psychosocial factors. Physicians should carefully evaluate psychotic symptoms, such as auditory hallucinations, not only during the postpartum period but also throughout the course of pregnancy.

## Abstract 2

The present report is the first report of a case of obvious auditory hallucinations and hyperemesis gravidarum at the same time in a pregnant woman who had not been diagnosed with any psychiatric disorder. We administered an antipsychotic agent, perospirone, to treat the auditory hallucinations, which disappeared, although the hyperemesis gravidarum persisted until childbirth. After childbirth, perospirone treatment was discontinued, and her auditory hallucinations did not relapse.

## 1 BACKGROUND

Perinatal psychiatric disorders, such as postpartum depression or puerperal psychosis, are well known.^1^, ^2^ However, psychiatric symptoms rarely occur during pregnancy due to various factors.^3^ We report a pregnant patient who experienced auditory hallucinations only while suffering from hyperemesis gravidarum. She had no underlying psychiatric disease. The patient gave written consent for the publication of this case report.

## 2 CASE PRESENTATION

The patient was a 24‐year‐old pregnant woman (20 weeks of pregnancy) who suffered from auditory hallucinations. She had never been diagnosed with any impairment in intelligence or communication skills and had no adverse childhood experiences such as abuse. She had never been diagnosed with epilepsy or any other physical illness. She had been drinking small amounts of alcohol twice per week. She also had no history of smoking or using other drugs such as cannabis or stimulant drugs. Although she was naturally nervous, she had no history of psychiatric disorders. When she was 22 years old, she became pregnant for the first time. She was admitted to the obstetrics clinic in our hospital twice for severe hyperemesis gravidarum. Her family blamed the condition on her physical and mental fragility. After admission, she developed mild auditory hallucinations of strangers blaming her. However, she was able to distract herself and not worry about the existence of the auditory hallucinations by talking to others such as the nurses or other patients. After her first child was born, the auditory hallucinations disappeared spontaneously, and her mental condition remained stable.

When the patient became pregnant again at the age of 24 years, she developed general malaise, insomnia, and vomiting during the early stage, similar to the first trimester of her first pregnancy. Starting at 15 weeks of pregnancy, she heard the doorbell when there was no one at the door and her cellphone ringtone even though it was switched off. From 20 weeks of pregnancy, she started to hear a voice criticizing her actions, even though there was no one nearby. Therefore, she visited the Department of Psychiatry in our hospital for psychiatric evaluation and treatment. She seemed to tire easily; however, her communication was smooth and did not show evidence of thought disturbances. The malaise, insomnia, and vomiting associated with hyperemesis gravidarum were more severe than in her first pregnancy. However, obvious depressive mood and loss of the sensation of pleasure were not observed. She said that the auditory hallucinations were clearer and longer than during her first pregnancy. She would suddenly hear a stranger's voice criticizing her several times a day, as well as voices of performers speaking accusatory words about her while she was watching television. The auditory hallucinations had become a distraction even when talking to someone. There was no obvious disturbance of consciousness, and no obvious neurological findings such as loss of tendon reflexes, gait disturbance, or eye movement disturbance were observed. There were no obvious abnormal findings in the blood test. Magnetic resonance imaging and electroencephalogram could not be performed because she was unable to rest for a long time due to hyperemesis gravidarum. At the first visit, she was prescribed zolpidem (5 mg/day) for her insomnia. The insomnia improved; however, moderate auditory hallucinations persisted. Then, we administered 4 mg/day of an atypical antipsychotic agent, perospirone, to treat the auditory hallucinations. Subsequently, the frequency of the auditory hallucinations decreased. When the dose of perospirone was increased to 12 mg/day, her auditory hallucinations disappeared. In the final month of pregnancy (36 weeks), the patient's auditory hallucinations temporarily relapsed due to the increase in stress and anxiety. Then, when we increased the dose of perospirone to 16 mg/day, her hallucinations disappeared again. However, her hyperemesis gravidarum persisted. The patient safely gave birth at 39 weeks. After childbirth, we discontinued perospirone treatment. Her mental condition was stable, and she had a normal daily life without relapse of auditory hallucinations.

## 3 DISCUSSION AND CONCLUSION

This patient, who had not been previously diagnosed with any psychiatric disorder, developed auditory hallucinations only during pregnancy. Although she suffered from hyperemesis gravidarum until she gave birth, her auditory hallucinations disappeared after she started taking an antipsychotic agent. After childbirth, her auditory hallucinations never relapsed, even though the medication was stopped. Based on her clinical course, she was diagnosed with a psychotic disorder due to another medical condition, according to the Diagnostic and Statistical Manual of Mental Disorders, 5th Edition. We concluded that her transient auditory hallucinations developed only during pregnancy, and she was not diagnosed with a conventional psychiatric disorder. Mannion et al reported that 81% of pregnant women experienced at least one delusional‐like experience, and 76% of pregnant women without a history of psychosis had hallucination‐like experiences.^4^ However, this report did not examine the presence or influence of hyperemesis gravidarum or its association with hallucinations and delusions during pregnancy. To the best of our knowledge, the present report is the first case of hyperemesis gravidarum in pregnancy acting as “another medical condition” causing transient auditory hallucinations.

Human chorionic gonadotropin has been reported as a diagnostic marker of hyperemesis gravidarum, and free thyroxine levels are correlated with hyperemesis gravidarum.^5^, ^6^ Furthermore, various other hormones, such as leptin, progesterone, and adrenal cortical hormones, have been implicated in the etiology of hyperemesis gravidarum.^7^ Changes in these hormones could have induced the transient excessive release of or hypersensitivity to dopamine in this case. The frequency of auditory hallucinations has been reported to be higher in female patients with schizophrenia than in male patients, and an association with changes in estrogen has been indicated.^8^ Individual susceptibility or hypersensitivity to changes in female sex hormones could affect the development of psychiatric disorders during pregnancy. Furthermore, because the auditory hallucinations were immediately improved after initiation of perospirone treatment, the underlying mechanism could have involved excessive dopamine release. Perospirone is a second‐generation antipsychotic used in Japan and acts as a serotonin (5‐HT)_1A_ receptor partial agonist, 5‐HT_2A_ receptor inverse agonist, and dopamine (D)_2_, D_4_, and α_1_‐adrenergic receptor antagonist.^9^ Compared with haloperidol, perospirone showed no apparent difference in efficacy in terms of the Positive and Negative Syndrome Scale (PANSS) total score and the positive and general subscale scores, while perospirone was superior for negative symptoms.^10^ Perospirone produced fewer extrapyramidal symptoms than other antipsychotics.^10^ In this case report, no obvious adverse effects during the course of treatment with perospirone were observed. Annagür et al reported that the prevalence of any mood disorder was 14.9% and that of any anxiety disorder was 25.5% in women with hyperemesis gravidarum in the first trimester of pregnancy; however, this patient developed auditory hallucinations, and psychosis was not reported in that study.^11^

The rate of depression during the first trimester of pregnancy is similar to that observed in the general female population, whereas the rates during the second and third trimesters are nearly double the rate in the general female population.^3^ Recently, we reported that the prevalence of depression during pregnancy is 14.0% in the second trimester and 16.3% in the third trimester in Japanese women.^12^ Furthermore, it was reported that the mean Edinburgh postnatal depression scale score was higher during pregnancy than during the postnatal period, and the severity and nature of the depressed mood were not observed to change after childbirth.^13^

Personal characteristics and environmental factors are closely related to the onset and aggravation of hyperemesis gravidarum, and patients with serious cases of emesis gravidarum tend to develop various psychiatric symptoms.^14^ We also suspected that psychosocial factors such as the patient's original nervous disposition and inappropriate behaviors of her family might have affected the development of auditory hallucinations when hyperemesis gravidarum was aggravated in this case. In summary, we suggest that auditory hallucinations occurred as a result of the interaction of biological factors, such as changes in female hormones due to pregnancy or hyperemesis gravidarum, and psychosocial factors, such as her original nervous disposition and the lack of familial support.

We report a case of auditory hallucinations developing in a healthy pregnant woman with hyperemesis gravidarum. Clinicians, especially obstetricians, who examine pregnant women should carefully monitor the onset of psychiatric symptoms such as auditory hallucinations during pregnancy because various factors could affect the mental state of pregnant women.

## CONFLICT OF INTEREST

The authors declare that they have no competing interests.

## AUTHORS' CONTRIBUTIONS

HO treated the patient and acquired data and wrote the manuscript. NYF and KS supervised the work, and substantively revised it. All authors read and approved the final manuscript.

## APPROVAL OF THE RESEARCH PROTOCOL BY AN INSTITUTIONAL REVIEWER BOARD

The ethics committee is not required to review case reports.

## INFORMED CONSENT

The patient has consented in a written form to the submission of the case report for submission to the journal.

## Data Availability

The data are not publicly available due to privacy restrictions.
